# Roles of PRR-Mediated Signaling Pathways in the Regulation of Oxidative Stress and Inflammatory Diseases

**DOI:** 10.3390/ijms22147688

**Published:** 2021-07-19

**Authors:** Pengwei Li, Mingxian Chang

**Affiliations:** 1 State Key Laboratory of Freshwater Ecology and Biotechnology, Key Laboratory of Aquaculture Disease Control, Institute of Hydrobiology, Chinese Academy of Sciences, Wuhan 430072, China; pengweisuo@163.com; 2 Innovation Academy for Seed Design, Chinese Academy of Sciences, Wuhan 430072, China; 3 University of Chinese Academy of Sciences, Beijing 100049, China

**Keywords:** pattern recognition receptors, signaling pathways, reactive oxygen species, oxidative stress, inflammatory diseases

## Abstract

Oxidative stress is a major contributor to the pathogenesis of various inflammatory diseases. Accumulating evidence has shown that oxidative stress is characterized by the overproduction of reactive oxygen species (ROS). Previous reviews have highlighted inflammatory signaling pathways, biomarkers, molecular targets, and pathogenetic functions mediated by oxidative stress in various diseases. The inflammatory signaling cascades are initiated through the recognition of host cell-derived damage associated molecular patterns (DAMPs) and microorganism-derived pathogen associated molecular patterns (PAMPs) by pattern recognition receptors (PRRs). In this review, the effects of PRRs from the Toll-like (TLRs), the retinoic acid-induced gene I (RIG-I)-like receptors (RLRs) and the NOD-like (NLRs) families, and the activation of these signaling pathways in regulating the production of ROS and/or oxidative stress are summarized. Furthermore, important directions for future studies, especially for pathogen-induced signaling pathways through oxidative stress are also reviewed. The present review will highlight potential therapeutic strategies relevant to inflammatory diseases based on the correlations between ROS regulation and PRRs-mediated signaling pathways.

## 1. Introduction

Pattern recognition receptors (PRRs) are the primary sensors for host cell-derived damage associated molecular patterns (DAMPs) and microorganism-derived pathogen associated molecular patterns (PAMPs) and can initiate downstream inflammatory response. It has been acknowledged that inflammatory response is a double-edged sword, which can eradicate the invading pathogen and repair tissue damage sometimes but cause acute and chronic inflammatory diseases at other times. For example, in the context of lethal influenza A virus (IAV) infection, the NLRP3 inflammasome played a crucial role in virus clearance during early-stage infection, however it induced harmful inflammatory response that contributed to pathogenesis and mortality during late-stage infection [1]. Activation of the NLRC4 inflammasome is important for the pulmonary clearance of flagellated *Legionella* spp. [2,3], but NLRC4 inflammasome activation and subsequent IL-18 production contribute to gastric inflammation and dampen host defenses during *Helicobacter pylori* infection [4]. Furthermore, NLRC4 inflammasome mutations have been linked to autoinflammatory diseases with recurrent macrophage activation or enterocolitis syndromes [5,6]. Evidence has been increasingly emerged that oxidative stress plays a crucial role in regulating inflammatory response balance. ROS (reactive oxygen species) and RNS (reactive nitrogen species) are primary free radicals species which contain one or more unpaired electrons, and mainly included the superoxide radical anion (O_2_•^−^), hydroxyl radical (OH), nitric oxide (NO), peroxyl radicals, as well as non-radical species such as hydrogen peroxide (H_2_O_2_), singlet oxygen ((1)O_2_), hypochlorous acid (HOCl), and peroxynitrite (ONOO^−^) [7,8,9]. Free radicals are originated from the mitochondrial electron transport chain, nicotinamide adenine dinucleotide phosphate (NADPH) oxidase, xanthine oxidase, dual oxidase, and nitric oxide synthases [8]. Moreover, the beneficial effects of moderate free radicals for antibacterial and antiviral responses have been emphasized, ROS and/or RNS directly showed oxidative damage to microbes [10,11], as well as indirectly mediated signal transduction cascades such as autophagy, mTOR kinase, apoptosis, and immune pathways [12,13]. For example, after TLRs recognition of PAMPs and DAMPs, ROS generation enhanced TLR-stimulated production of proinflammatory cytokines as positive feedback [12,14]. Otherwise, ROS are necessary for NLRP3 inflammasome activation. The accumulation of ROS concentration following cellular stress leads to the dissociation of thioredoxin-interacting protein (TXNIP) from oxidized thioredoxin-1 (Trx-1), subsequent association of TXNIP with NLRP3 (via its NBD and/or LRR domain) and thereby NLRP3 activation [15,16]. However, intracellular overproductions of ROS or RNS can lead to oxidative stress, which result from the imbalance of ROS generation and consumption, and further trigger proinflammatory cascade and chronic inflammation or autoimmune diseases. In diabetes mellitus, a variety of factors including environmental factors, dietary components and/or inappropriate immune responses to host antigens trigger an autoimmune attack against the pancreatic β-cell, and ROS release from phagocytes can lead to a failure to resolve inflammation and the amplification of the autoimmune attack against the β cells, which contribute to the progression of the disease [17]. In chronic kidney disease, oxidative stress, mainly mediated by NADPH oxidases in renal monocytes, endothelial cells, and mesangial cells, could accelerate kidney disease progression and associate with complications such as hypertension, atherosclerosis, inflammation, and anemia [18,19]. In the current review, we summarized and highlighted ROS production mediated by PRR-dependent pathways, the relationships and internal molecular mechanisms between ROS imbalance and inflammatory diseases, as well as therapeutics strategies against inflammatory diseases based on the correlations between ROS regulation and PRRs-mediated signaling pathways.

## 2. Oxidative Stress Mediated by PRR-Dependent Pathways

ROS are either free radical molecules or nonradicals. The generation and biological functions of free radicals have been reviewed by Jha et al. [19]. In briefly, the superoxide radical anion is formed by NADPH oxidases, xanthine oxidase, or mitochondrial electron transport chain, and then transformed either to H_2_O_2_ by spontaneous and/or superoxide dismutase (SOD)-catalyzed dismutation, or to form highly ONOO^−^ via an interaction with NO. At high concentrations, ROS are important mediators for protein modification, lipid peroxidation process, DNA damage, and so on [8,20,21]. Here, we reviewed oxidative stress mediated by PRR-dependent pathways, including TLRs, NLRs, and RLRs signaling pathway.

### 2.1. ROS Generation Mediated by TLRs Activation

TLRs are type I transmembrane proteins composed of an extracellular leucine-rich repeat domain for DAMP/PAMP recognition, a transmembrane domain, and a cytoplasmic Toll/IL-1 receptor (TIR) homology domain for signal transduction. TLRs divide into two types, among these conserved TLRs, the plasma membrane located TLRs (TLR1, TLR2, TLR4, TLR5, TLR6, TLR10 and TLR11) sense bacterial lipoproteins, peptidoglycans, flagellin and lipopolysaccharide, while the endosomal TLRs (TLR3, TLR7, TLR8, TLR9, and TLR13) detect viral double-stranded RNAs, single-stranded RNAs, DNAs, or unmethylated CpG-DNA [22]. After DAMPs or PAMPs recognition via LRR domain, the cytoplasmic TIR domains of TLRs recruit signaling adaptors MyD88, TIRAP, TRAM, and/or TRIF, then various kinases (IRAK4, IRAK1, IRAK2, TBK1, and IKK1) and ubiquitin ligases (TRAF6 and pellino 1) are recruited and activated, which lead to culminating in the engagement of NF-kB, type I interferon, p38 MAPK, and JNK MAPK pathways, and generating proinflammatory cytokines including tumor necrosis factor α (TNF-α), interleukin (IL)-1β, IL6, and chemokines [23,24].

TLRs were tested the ability for inducing ROS generation in RAW 264.7 macrophages, but only cell-surface TLR1, TLR2, and TLR4 could trigger ROS production upon stimulated with LPS (a TLR4 agonist), Pam3CK4 (TLR1 and TLR2 agonist), and LTA (a TLR2 agonist), whereas stimulation of endosomal TLRs (TLR3/7/8/9) failed to induce ROS [25]. Accumulating evidences have shown that the NADPH oxidase (NOX) activation, existing as seven distinct isoforms (NOX1–NOX5 and the dual oxidases DUOX1 and DUOX2), may be the key factor linking TLR2/TLR4 and ROS generation possibly via several mechanisms including I) the induced expression of NOX; II) the stimulated assembly of the NOX subunits; III) direct interactions between NOX and the TIR domain of TLRs at the cell membrane [26,27]. NOX enzymes transfer electrons from NADPH to oxygen for generating O_2_•^−^ and finally converting to H_2_O_2_ and O_2_ in cell [28]. Among seven NOX isoforms, p22^phox^ is a membrane subunit associated with NOX2, p47^phox^ thought to be the organizer subunit of NOX2, NOXO1 for the organizer subunit of NOX1 and NOX3, p67^phox^ for the activator subunit of NOX2, and NOXA1 for the activator subunit for NOX1 [29]. The recruitments of p22^phox^, p67^phox^, p47^phox^, and Rac protein are necessary for NOX activation [30]. In tumor tissues, TLR/MyD88 signaling and the COX-2/PGE2 pathway are required for induction of inflammatory cytokines such as TNF-α. However, TNF-α signaling induces activation of NOX1/ROS signaling, which lead to increase ROS production and promote tumorigenesis [31]. Similar results have shown that NOX1-deficient mice fail to generate ROS in response to Pam3CSK4 stimulation, which indicate that NOX1 is essential for TLR2-dependent production of ROS [32].

**Figure 1:**
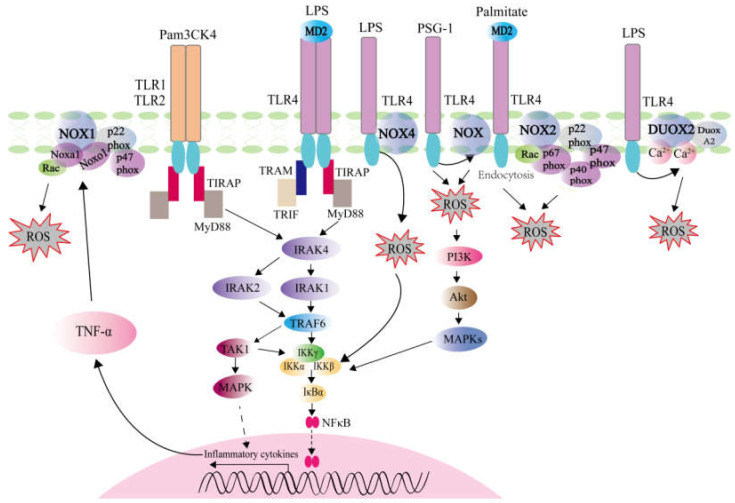
ROS generation induced by TLR activation. TLR1, TLR2, and TLR4 recruit MyD88 and TRAF6 to activate the NF-κB and MAPK pathways under the action of the ligand Pam3CSK4 or LPS, which can trigger the expressions of inflammatory cytokines such as TNF-α. NOX1 complex is activated by TNF-α signaling, which further leads to increased ROS production. The monomeric TLR4–MD2 complex in response to palmitate treatment generates ROS by NOX2 complex. The direct interaction of TLR4 with NOX4 is essential for LPS-mediated ROS generation and NF-κB activation. TLR4 is the major receptor involved in specific binding of PSG-1 to macrophages, and PSG-1 exerts its effects on macrophage activation via the TLR4/ROS/PI3K/Akt/MAPKs/NF-κB signal pathway. TLR4 activation also leads to increased ROS production through DUOX2.

The correlation between TLR4 and NOX-dependent ROS was investigated. In the LPS-stimulated non-small cell lung cancer (NSCLC) cells, ROS level was increased and significantly abolished by NOX1 inhibitor ML171. Significantly, the increased NOX1 expression in tumor tissues was positively correlated with the expression level of TLR4 and the clinical stages in NSCLC patients [33]. The TLR4 dependence of LPS-induced NOX1 expression was also confirmed in the Raw264.7 cells, peritoneal macrophages, and mice [34,35]. Furthermore, the mechanism of palmitate-mediated ROS generation via NOX2 was demonstrated, and it was found that endocytosis of the TLR4–MD2 complex generated ROS by a MyD88/TRIF-independent in human macrophages, which might be related with pathogenesis of non-alcoholic fatty liver disease (NAFLD) in human patients [36]. Conversely, NOX2 expressed in macrophages and neutrophils limited the transcriptions of TLR4 and TNF-α induced by LPS [37]. NOX4 is a protein related to gp91^phox^ (NOX2) which belongs to transmembrane flavocytochrome b components. The COOH-terminal region of NOX4 directly interacted with TIR of TLR4, and the interaction between TLR4 and NOX4 contributed to the production of ROS and NF-κB activation in response to LPS stimulation in HEK293T cells [38]. In the regulation of *Ganoderma atrum* polysaccharide (PSG-1) stimulated TNF-α production, TLR4-mediated ROS production act as upstream of PI3K/AKT/MAPKs/NF-KB signaling pathway [39]. Recently, a report showed the intersection of TLR4 signaling, epithelial redox activity and the microbiota in colitis-associated neoplasia. It was found that colonic epithelial cells (CECs) respond to Gram-negative bacterial challenge by inducing DUOX2-mediated release of ROS in a TLR4-dependent fashion [40]. In summary, NOX enzymes act downstream of TLRs signaling pathway, which are associated with ROS generation (Figure 1).

### 2.2. ROS Generation Mediated by NLRs Activation

NLRs distribute in cytoplasm and have conversed structure in mammals and plants: a N-terminal domain for interaction with downstream adaptor proteins, a central nucleotide-binding and oligomerization domain for activation, and multiple C-terminal leucine-rich repeat domains for ligand recognition [41]. NLRs can be divided into at least four subfamilies distinguished by N-terminal domain: NLRA containing an acidic transactivation domain, NLRB containing baculoviral inhibitory repeat, NLRC containing caspase recruitment and activation domain, and NLRP containing pyrin domain [42]. Most NLRs act as PRRs, which mainly sense Gram-positive bacteria γ-D-glutamyl-meso-diaminopimelic acid, Gram-negative bacteria peptidoglycan component, viral RNA, and so on. The activated NLRs have various functions and can be divided into four broad categories: signaling transduction, inflammasome formation, transcription activation, and autophagy [43].

#### 2.2.1. ROS Generation Mediated by NLRs Involving in Signaling Transduction and Autophagy

**Figure 2:**
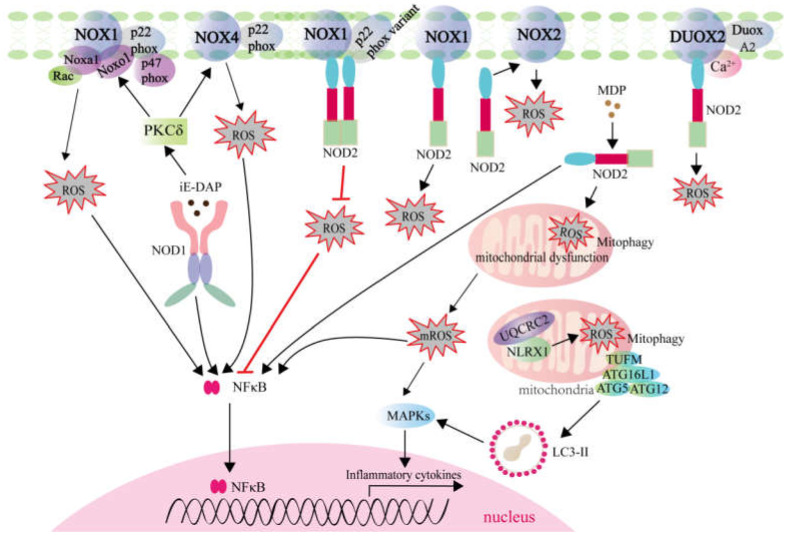
ROS generation mediated by NLRs involved in signaling transduction and autophagy. NOD1 and NOD2 are two members of NLRC subgroup, which are located at cytoplasm sensing γ-D-glutamyl-meso-diaminopimelic acid (iE-DAP) and muramyl dipeptide (MDP), respectively. NOD1/NOD2-induced ROS is correlated with NOXs activation, including NOX1, NOX2, NOX4, and DUOX2. However, NOX1 and p22phox variants attenuate ROS production and impair innate immune defense mechanisms via NOD2. Furthermore, NOD2 activation induces ROS contributing to mitochondrial dysfunction in skeletal muscle cells. Different from NOD1 and NOD2, NLRX1 is a mitochondria-associated protein, and contributes to the link between mitochondrial ROS (mROS) generation and innate immune responses.

NOD1 and NOD2 are two well-known members of the NLRC subgroup, and act as positive regulatory NLRs. The unique negative regulatory NLRs are currently composed of three members, namely NLRP12, NLRX1, and NLRC3. Each of these NLRs modulates diverse signaling pathways, including nuclear factor kappa B (NF-kB), mitogen-activated protein kinase (MAPK), type I IFN response, autophagy and so on [44]. Among them, NOD1, NOD2, and NLRX1 are involved in the generation of ROS (Figure 2).

In neutrophils of periparturient dairy cows, the role of NOD1 in the production of ROS was investigated. Although NOD1 activation enhanced phagocytosis and ROS production in the isolated cell populations compared to the unstimulated cells, NOD1-activated neutrophils showed reduced phagocytosis and ROS generation in periparturient cows compared with mid-lactating cows [45]. In adipocytes from Male C57BL/6 mice, NOD1 activation also induced ROS generation and oxidative stress. It was found that PKCδ-NOX1/4 signaling was involved in the NOD1-mediated ROS generation [46].

Compared with the reports involved in NOD1, more studies revealed the role and mechanisms of NOD2 in ROS generation. First, direct physical interactions have been demonstrated for NOD2 and NOX family members. NOD2 was found to physically interact with NOX1, which led to increased production of ROS in osteoclasts [47]. NOD2 also interacted and colocalized with DUOX2 in HEK293 cells. In primary murine intestinal epithelial cells isolated from colon of NOD2-knockout mice (NOD2^−/−^), no response to MDP stimulation with regard to intracellular ROS levels was observed. Different from that from NOD2^−/−^ mice, increased ROS generation was detectable in stimulated tissue samples from WT mice. Further studies revealed that NOD2 facilitated ROS generation in intestinal epithelial cells involving DUOX2 activation [48]. In addition to NOX1 and DUOX2, p22^phox^ was identified as a novel NOD2 interactor. However, the attenuated ROS production by NOX1 and p22^phox^ variants was found to impair the NOD2-mediated immune responses [49]. Although the regulatory mechanisms of NOD2 for activating NOX2 are needed to address, NOX2 has been confirmed to be an important source for NOD2-induced ROS production [50]. NOD2 also played a pivotal role in MDP-induced COX-2/NOX4/ROS signaling pathway in human umbilical vein endothelial cells (HUVECs), which was a novel regulatory mechanism for ROS generation mediated by NLRs [51]. Second, several studies suggested that mitochondria contributed to NOD2 activation-induced ROS production. In muscle cells, NOD2 activation-induced ROS are mitochondria-dependent [52]. Mitochondria are a major source of intracellular ROS, and mitophagy alterations can lead to elevated ROS levels. In intestinal stem cells, NOD2 activation led to mitophagy, therefore balancing intracellular ROS levels [53]. Furthermore, generation of the bactericidal ROS via NOD2-dependent pathway has been shown for intracellular bacteria such as *Listeria monocytogenes* and *Acinetobacter baumannii* [48,54].

NLRX1 is the only member of NLR family that translocates to the mitochondria. Overexpression of NLRX1 enhanced mitochondrial ROS (mROS) generation in response to cytokine TNF-α and poly(I:C) stimulation, *Shigella* and *Chlamydia trachomatis* infection [55]. On the contrary, knockdown of NLRX-1 blocked rhinovirus-induced mROS or attenuated ATP-induced mROS [56,57]. In terms of mechanism, NLRX1 can modulate mROS during infection possibly through interaction with UQCRC2, a matrix-facing protein of the respiratory chain complex III (bc1 complex) [58]. NLRX1 augmented the TNF-α induced ROS by regulating the activities of mitochondrial respiratory Complex I and Complex III [59], and potentiated mROS generation in response to cisplatin exposure via autophagic cell death pathway [60].

#### 2.2.2. ROS Generation Mediated by NLRs Involving in Transcription Activation

NLRC5 and NLRA (CIITA) are the NLRs involved in transcription activation of MHC class I and MHC class II genes [43]. At present, only three reports revealed the inconsistent role of NLRC5 in ROS generation. In the NLRC5-knockout and -knockdown cardiac muscle cells challenged with LPS, no obvious effect was observed for ROS production [61]. However, overexpression of NLRC5 caused significant decrease in ROS levels [62], and NLRC5 silencing was associated with increased ROS levels under oxygen-glucose deprivation/reperfusion (OGD/R) [63]. The exact mechanism of NLRC5 on the regulation of ROS generation remains to be clarified.

#### 2.2.3. ROS Generation Mediated by NLRs Involving in Inflammasome Formation

**Figure 3:**
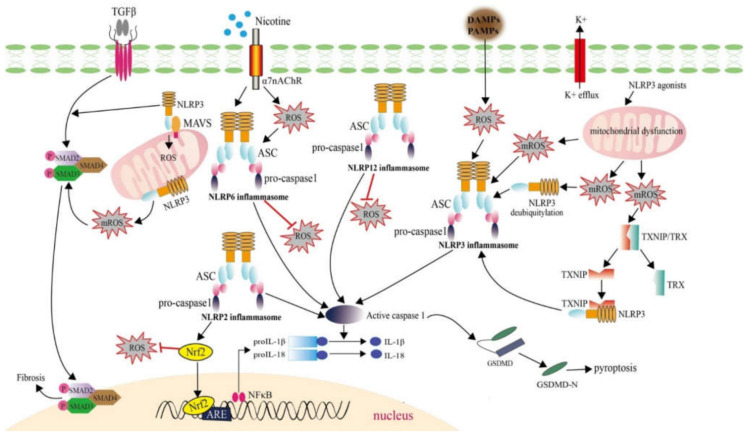
ROS generation mediated by NLRPs and their roles in regulating NLRP inflammasome activation. NLRP2, NLRP3, NLRP6, and NLRP12 inflammasome are involved in ROS generation. Among them, the NLRP2, NLRP6, and NLRP12 inflammasomes are negative regulators for ROS generation, and the NLRP2 inflammasome mediates ROS generation through activating Nrf2 signaling. NLRP3 acts both upstream and downstream of ROS in an inflammasome-dependent as well as an inflammasome-independent pathway. On the one hand, ROS activation of NLRP3 inflammasomes have been demonstrated to associate with mROS and DAMP/PAMP induced ROS. On the other hand, inflammasome-independent NLRP3 regulates mROS in cardiac fibroblast under TGF-β1 stimulation and plays important role in mROS production by binding to MAVS in renal tubular cells.

Inflammasome formation is triggered by various PAMPs derived from bacteria (including pore-forming toxins, lethal toxins, flagellin, muramyl dipeptide MDP, RNA, and DNA), viruses, fungus (β-glucans, hyphae, mannan and zymosan) and protozoa (hemozoin), by self-derived DAMPs (such as ATP, cholesterol crystals, monosodium urate, glucose, amyloid β, and hyaluronan), and by environment-derived activators [43]. Eight NLRs (NLRP1, NLRP2, NLRP3, NLRP6, NLRP7, NLRP12, NLRC4, and NAIP) can form inflammasome after directly recognition PAMPs/DAMPs or downstream of TLRs signaling pathway, and next after recruiting apoptosis-associated speck-like protein containing a CARD (ASC) and/or pro-caspase-1, which activate caspase-1 and promote pyroptosis progress via the secretion of mature IL-1β/IL-18 and gasdermin D activation [64,65]. Among them, NLRP2, NLRP3, NLRP6, and NLRP12 have been found to be involved in the generation of ROS (Figure 3).

NLRP2 is a cytoplasmic protein, which is activated by ATP and has a critical role in the astrocyte innate immune response [66]. In the liver of high-fat diet (HFD)-fed mice, NLRP2 deletion accelerated inflammation and oxidative stress. In the primary hepatocytes treated with palmitate, it was found that NLRP2 deletion meditated inflammation and ROS generation through repressing NF-E2-related factor 2 (Nrf2) pathway [67].

The best characterized inflammasome is NLRP3 inflammasome. The earring-shaped inactive NLRP3 has a N-terminal pyrin domain which interacts with pyrin domain of ASC [68,69]. Three molecular mechanisms have been proposed to clarify the activation of NLRP3 inflammasome: pore formation and potassium (K1) efflux, lysosomal destabilization and rupture, and ROS generation [70,71,72]. The ROS sources required for NLRP3 activation possibly come from two parts: mROS and DAMP/PAMP induced ROS. First, NLRP3 deubiquitylation is regulated by mROS [73]. NLRP3 Inflammasome activation and IL-1β/IL-18 mature is triggered by mROS induced by ATP, LPS, or ethanol in astrocytes and is abolished by TLR4 KO [74]. The relationship of NLRP3 inflammasome activation and TLR2/TLR4-mROS axis was also reported in PGN/LPS induced macrophages [75]. It has been reported that TXNIP was dissociated from TRX at a high concentration of H_2_O_2_ or mROS, interacted the NACHT/LRR domain of NLRP3 and mediated NLRP3 inflammasome activation [16,76]. On the other hand, almost all NLRP3 agonists can result in mitochondrial dysfunction and the robust generation of ROS [77], which further facilitated activation of the NLRP3 inflammasome [65]. NLRP3 also acts as an inflammasome-independent way. In renal tubular cells, the deletion of NLRP3 attenuated mROS production. In response to unilateral ureter obstruction model (UUO), NLRP3^−/−^ mice showed less ROS injury than WT mice. NLRP3 in renal tubular cells played important role in ROS generation by binding to MAVS after hypoxic injury [78]. Similarly, silencing of the NLRP3 by shRNA resulted in reduced generation of ROS in HK-2 cells under high glucose conditions [79]. In response to TGFβ signaling in cardiac fibroblasts, NLRP3 also regulated mROS production to augment Smad signaling, which ultimately led to chronic disease [80]. However, a report also showed that NLRP3 was not required for mROS generation by LPS and ATP treatment in macrophages [81]. Therefore, ROS-mediated NLRP3 inflammasome activation and NLRP3-induced ROS production suggest a dual function for NLRP3 both upstream and downstream of ROS.

NLRP6 (originally termed PYPAF5) can recruit ASC and the inflammatory caspase-1 or caspase-11 to form an inflammasome, which is critical in tissue homeostasis and disease modulation [82,83]. NLRP12 functions as a regulator of inflammatory signaling, and a negative regulator for many canonical and non-canonical signaling including the NF-κB and MAPK signaling pathways [84,85]. Similar to NLRP3, NLRP6 and NLRP12 can exert its function in an inflammasome-dependent as well as an inflammasome-independent pathway [82,86]. Different from NLRP3, NLRP6 and NLRP12 negatively regulated the production of ROS. It was found that higher ROS production was generated by NLRP6 deletion or NLRP12 mutation [87,88]. However, the negative regulatory mechanisms of NLRP6 and NLRP12 on the regulation of ROS generation are unclear at present.

### 2.3. ROS Generation Mediated by RLRs Activation

Retinoic acid-inducible gene I (RIG-I)-like receptors (RLRs) are key sensors for viral RNA in the cytoplasm and mediating the production of type I interferons (IFNs) and IFN-stimulated genes (ISGs). The RLRs signaling pathway is well-charactered that RLRs detect viral RNA and combine with mitochondrial antiviral-signaling protein (MAVS) via CARD-CARD interaction. MAVS then activates TANK-binding kinase 1 (TBK1) and IκB kinase-ε (IKKε), which subsequently activate IRF3 and IRF7 transcription factor, and induce the expression of type I IFNS and ISGs. RLRs include three members: RIG-I, melanoma differentiation-associated protein 5 (MDA5), and laboratory of genetics and physiology 2 (LGP2). It is clear that infections with almost all major virus families such as double-stranded DNA (dsDNA), single-stranded DNA (ssDNA), double-stranded RNA (dsRNA), ssRNA (+), ssRNA (–), ssRNA (reverse transcribing), and dsDNA (reverse transcribing) virus are recognized by RLRs in mammals [89].

**Figure 4:**
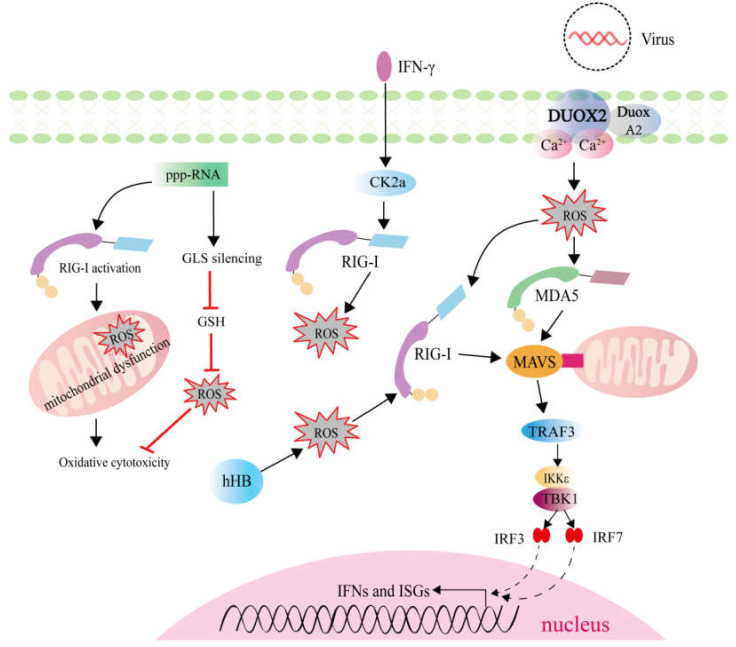
ROS generation involved in RLRs signaling pathway. RIG-I and MDA5 are key sensors for viral RNA in the cytoplasm. RIG-I activation induced by ppp-RNA or IFN-γ can induce ROS accumulation. ROS induced by hemoglobin subunit beta (HB) and DUOX2 can trigger the activation of RLRs signaling pathway.

In response to viral infection, activation of the innate immune system can induce the production of ROS. In mice, in vivo RIG-I stimulation induced endothelial dysfunction, and was associated with a significant increase in vascular ROS [90]. In IFNγ-treated glioma cells, inhibiting RIG-I through siRNA mediated knockdown elevated ROS levels, but IFNγ itself had no effect on ROS production, which confirmed that RIG-I mediated the regulation for ROS generation [91]. 5′-Triphosphate RNA (ppp-RNA), a specific ligand of RIG-I, could induce ROS accumulation via RIG-I activation and mediate ROS elimination via glutaminase (GLS) silencing [92]. However, similar to NOD2 and NLRP3, RIG-I also functions upstream and downstream of ROS (Figure 4). Hemoglobin is an important oxygen-carrying protein, which plays crucial roles in the host resistance against pathogen infection. The hemoglobin subunit beta (HB) could induce ROS generation to promote RIG-I-mediated signaling via enhancement of K63-linked RIG-I ubiquitination [93]. In mouse nasal mucosa and human nasal epithelium, DUOX2-derived ROS are necessary for triggering the induction of RIG-I and MDA5 to resist against influenza A virus (IAV) infection [94]. Different from RIG-I, the effects of MDA5 and LGP2 in regulating ROS generation remain unknown. More researches are needed to investigate the crosstalk and mechanisms for ROS-dependent activation of RLRs signaling and RLRs-mediated ROS production, especially for MDA5, LGP2, and the ligands of RLRs.

Mitochondria are important for cell energy metabolism and also participant in innate and adaptive immune responses. As mentioned above, mitochondria ROS is one of main source of ROS generation and important for NLRP3 inflammation activation. The crosstalk between mROS and RLRs signaling pathway have been reported, and it was found that the inhibition of mitophagy by atg5 deficiency enhanced the accumulation of mROS, which further amplify RLRs signaling [95]. Superoxide dismutase 2 (SOD2) is essential in radical scavenging, which plays a pivotal role in balancing the intracellular level of ROS. In SOD2-silenced cells, the higher level of mROS was observed. SOD2 deficiency-mediated mROS accumulation potently inhibited RLRs-induced innate immune responses through the regulation of NF-κB and IRF3 activation [96]. All these results demonstrate the role of mROS in mediating RLRs signaling pathway.

## 3. Therapeutic Targets for Suppressing Inflammatory Diseases Based on the Correlations between ROS Regulation and PRR-Mediated Signaling Pathways

### 3.1. Therapeutic Targets for Inflammatory Vascular Diseases

Diabetic cardiovascular diseases are characterized by progressive hyperglycemia, which results in excessive production of ROS. However, apoptosis is an early event involved in cardiomyopathy associated with diabetes mellitus. TLRs are activated in diabetic patients, and the increased ROS are also produced in various tissues under diabetic conditions. It was found that suppression of hyperglycemia-induced apoptosis by TLR4 is associated with attenuation of ROS production [97]. In macrovascular cells, hyperglycemia induced activation of TLR2 and TLR4 through NOX-mediated ROS production. The treatment of antioxidants such as apocynin and N-acetyl cysteine (NAC) could decrease high glucose-induced ROS levels as well as TLR2/TLR4 expression and downstream inflammatory biomediators [98]. P-Coumaric Acid (P-CA) is a member of hydroxycinnamic acid family, which exhibits antidiabetic and potent antioxidant effects. Oral administration of P-CA significantly improved diabetic nephropathy-induced histopathological abnormalities, reduced kidney TLR4 level, and attenuated oxidative stress [99]. As the increases in ROS production and TLRs activity are ameliorated with antioxidant strategies, targeting ROS/NADPH oxidase with small molecular inhibitors or TLR antagonists could be a promising strategy to reduce both ROS and TLR-mediated inflammatory diseases in diabetic vascular diseases.

The role of NLRP3 inflammasome in cardiovascular disease has been reported and confirmed to be involved in hyperhomocysteinemia (HHcy)-aggravated inflammation and atherosclerosis. Intracellular ROS participate in NLRP3 inflammasome activation in HHcy mice model and the high-fat diet (HFD)-induced atherosclerosis model using apolipoprotein E deficient (ApoE^−/−^) mice. Treatment with NAC in HHcy mice suppressed NLRP3 inflammasome activation and improved HHcy-induced atherosclerosis [100]. NAC also inhibited the ROS-mediated nicotine–NLRP3–ASC–pyroptosis pathway and nicotine-induced pyroptosis [101]. Peperomin E (PepE) is another antioxidant phytochemical. PepE treatment inhibited atherosclerosis development by suppressing NLRP3 inflammatory pathway in HFD-fed ApoE^−/−^ mice [102]. In cardiac microvascular endothelial cells (CMECs), an ROS scavenger EUK134 could dissociate TXNIP from NLRP3 and blocked the NLRP3 inflammasome activation [103]. Trimethylamine-N-oxide (TMAO) was identified as a risk factor for promoting atherosclerosis through inducing vascular inflammation. The mitochondrial ROS scavenger Mito-TEMPO was found to ameliorate TMAO-induced endothelial NLRP3 inflammasome activation and attenuate vascular inflammation through the SIRT3–SOD2–mitochondrial ROS signaling pathway [104]. Therefore, antioxidants and therapeutic targets of ROS scavengers could be a promising strategy to intervene ROS-NLRP3-dependent vascular inflammation and diseases.

### 3.2. Therapeutic Targets for Inflammatory Neurological Diseases

Parkinson’s disease (PD) is the second most common progressive neurodegenerative disorder, which is associated with neurobehavioral disorders, cognitive impairment, and autonomic dysfunction [105]. ROS production plays a key role in the pathogenesis of PD. Curcumin treatment, a polyphenolic compound isolated from *Curcuma longa* root, reduced ROS production and the level of TLR4. The anti-inflammatory action of curcumin was mediated via an inhibition of TLR4 and its downstream signaling pathway [106]. Tomentosin is an active compound isolated from the plant. In the PD mice, tomentosin treatment decreased the production of ROS, inhibited the TLR4/NF-κB signaling pathway, and prevented inflammation-mediated neuronal cell damage [107]. It was suggested that TLR4-mediated inflammation could be a potential target for PD therapy, and that the administration of curcumin and tomentosin could be potential agents in the inflammatory pathology of PD [106,107].

Oxidative stress and mitochondrial dysfunction are key pathophysiological processes for many chronic neurodegenerative diseases, including PD. Mitochondrial impairment in microglia can amplify NLRP3 inflammasome signaling and augment the neurodegenerative process in PD animal models [108]. The mitochondrial antioxidant mitoTEMPO could attenuate NLRP3 inflammasome activation via blockade of c-Abl and PKCδ activation. In the heightened microglial activation response, an exaggerated mROS/c-Abl/NLRP3 signaling axis was confirmed [109], which suggested that targeting the c-Abl-regulated NLRP3 inflammasome signaling offers a therapeutic strategy for PD. Astragaloside IV, the main active component isolated from *Astragalus membranaceus*, significantly lessened ROS generation in lipopolysaccharide (LPS)-induced BV2 microglia cells. Astragaloside IV might be the therapeutic drug for PD whose neuroprotective effects were largely dependent on the activation of the Nrf2 pathways and the suppression of NLRP3 inflammasome activation [110]. Furthermore, the Nrf2 signaling pathway is crucial for endogenous antioxidative system, and Nrf2 regulates the transcription of components of the glutathione and thioredoxin antioxidant systems [111]. In addition to PD, the protective effect of Nrf2 activation on Alzheimer’s Disease (AD) was also reported [112]. In short, Nrf2-related and c-Abl-regulated NLRP3 inflammasome signaling pathways may be used as potential therapeutic targets to prevent or slowdown the progression of neurodegenerative disorders.

### 3.3. Therapeutic Targets for Inflammatory Bowel Diseases

Inflammatory bowel diseases (IBD), including Crohn’s disease (CD) and ulcerative colitis (UC), are characterized by chronic relapsing intestinal inflammation. Bacterial sensing PRRs and autophagy dysfunction are involved in pathogenesis of IBD. NOD2 is the first identified susceptibility gene linked to CD, and specific TLR4 mutant alleles are associated with CD and UC [113,114]. At the intestinal epithelium, dysfunctional autophagy leads to accumulation of damaged mitochondria and ROS, and many autophagy-related genes such as Atg16L1 and IRGM, were also identified as susceptibility genes to CD [115,116]. In addition, the neutrophil cytosolic factor 2 (NCF2) and NCF4, which encode p67^phox^ and p40^phox^ components of the NOX2 NADPH oxidase, respectively, are susceptibility genes for CD [117,118]. Furthermore, in the case of very early onset IBD, the ROS-induced NOD2 activation via the p22^phox^-NOD2 interaction was abrogated by loss-of-function variants in the NADPH oxidase complex, which confirmed that missense variants in NOX1 and p22^phox^ are functionally linked to NOD2 [49]. Similar to the above results, the activity of NOX2 NADPH oxidase and its generated ROS are pivotal for inducing TLR4-activated autophagy [119]. All these results support that the associations of genetic variants in NOD2, TLR4, components of NADPH oxidases and autophagy genes play the significant roles in the pathogenesis of IBD.

The dysregulation of NLRP3 inflammasome contributes to IBD. Xi Lei San is a class of Chinese patent medicine, which is highly effective for treating IBD. Xi Lei San could repress apoptosis as well as ROS by reducing the activity of NLRP3 inflammasomes and autophagy [120]. Dimethyl fumarate (DMF) and 3-(2-oxo-2-phenylethylidene)-2,3,6,7-tetrahydro-1H-pyrazino-[2,1-a]isoquinolin-4(11bH)-one (compound 1), the Nrf2 activators, were found to ameliorate dextran sulfate sodium (DSS)-induced colitis (an animal model mimicking human IBD) via activating Nrf2 and inhibiting NLRP3 inflammasome [121,122]. Curcumin and procyanidin could alleviate DSS-induced colitis through inhibiting NLRP3 inflammasome activation [123,124]. Patchouli alcohol, a main active compound of traditional Chinese herb patchouli, showed improved therapeutic efficacy in a DSS-induced colitis by inhibiting MAPK/NF-κB pathway and NLRP3 inflammasome [125]. Therefore, antioxidants and therapeutic targets by targeting these NLRP3 inflammasome, MAPK/NF-κB and Nrf2 signaling pathways could be a strategy for the treatment of IBD.

Disturbances in the gut microbiota–host relationship have been associated with IBD. The benefits of the gut microbiota to the host’s physiology include nutrition, immune development, and host defense [126]. Increasing evidence has shown that some dietary nutrients from environmental factors and PRRs-mediated signaling pathways from host modulate the composition and metabolism of gut microbiota, which influence IBD onset [127,128,129,130,131,132,133]. Supplementation with 20 mg/kg melatonin (MT) by intraperitoneal injection could downregulate the colon ROS levels, and inhibit the changes of the intestinal microbiota and TLR2/TLR4 activities induced by restraint stress, which together mitigate psychological stress-induced colonic inflammatory responses [134]. Walnut oil, which has become a dietary supplement, could alleviate DSS-induced colitis by decreasing the ROS production, inhibiting NLRP3 inflammasome activation, and regulating the composition, relative abundance and SCFAs levels of gut microbiota [135]. All these results suggest that modulation of the gut microbiota through nutrient supplementation appears a promising potential therapeutic target for IBD.

## 4. Summary

As mentioned above, oxidative stress is the result of overproduction of oxidative free radicals and ROS. The cellular and molecular origins of ROS production and the roles of oxidative stress in pathophysiology and disease pathogenesis have been extensive reviewed. Previous report has illustrated the relevance of these interactions between stress proteins and cytoplasmic PRRs signaling in chronic inflammatory diseases, autoimmune disorders and cancer [136]. In the current review, we summarized the crosstalk between PRRs-mediated signaling pathway and ROS generation. Especially, ROS generation mediated by PRRs-dependent pathways, including TLRs, NLRs, and RLRs signaling pathways, was highlighted. Among these PRRs, NOD2, NLRP3, and RIG-I are the PRRs which function both upstream and downstream of ROS. The activation of TLR1, TLR2, TLR4, NOD1, NOD2, NLRX1, NLRP3, and RIG-I induced the production of ROS, but ROS generation was inhibited by NLRC5, NLRP2, NLRP6, or NLRP12 activation. Therefore, the present review firstly summarizes the similarity and difference of PRRs in regulating ROS production. Based on the correlations between ROS regulation and PRRs-mediated signaling pathways, the present review also highlights potential therapeutic strategies relevant to inflammatory diseases (Table 1). More researches are needed to investigate other PRRs-mediated ROS production, including the ligands of PRRs.

Increasing evidence has shown that targeting ROS/NADPH oxidase, NLRP3 inflammasome, MAPK/NF-κB signaling, Nrf2 signaling, autophagy, and mitochondrial bioenergetics provides potential therapeutic strategies for the prevention and treatment of various inflammatory diseases [15,137,138,139,140,141,142]. However, given the multifactorial etiology of inflammatory diseases and the limited success of antioxidant therapies achieved to date, therapeutic targets at single risk factor interventions are not enough to block the progression of inflammatory diseases. Understanding the molecular mechanisms behind the impact of PRR-mediated signaling pathways and their interactions with ROS production in the case of multifactorial etiology including the gut microbiota and dietary nutrients are crucial for developing new multiple pathway-integrated therapeutic strategies. Future research should investigate how nutrition and/or microbiota influence the interactions between PRR-mediated signaling pathways and ROS regulation. Moreover, the well-designed and targeted clinical studies should include investigating the safety and efficacy of these combinatorial strategies for preventing or treating oxidative stress-induced inflammatory diseases.

In regulating microbe-elicited ROS production during an antimicrobial response, many studies showed that host PRRs (mainly TLR2, TLR4, NOD2, NLRX1, and NLRP3) and redox modulation alleviated pathogen infection. For example, neutralization of TLR2 and blocking of host derived SOD and catalase facilitated the killing of *Staphylococcus aureus* within the bone marrow cells [143]. TLR4-NOX2 axis contributed to produce ROS, phagocytize, and kill bacteria such as *Mycobacterium tuberculosis* [11]. NOD2 in cooperative with DUOX2 protected against *Listeria monocytogenes* infection [48]. However only a few reports revealed the roles of host PRRs in regulating virus-elicited ROS production and infection. Significantly, the current coronavirus COVID-19 or severe acute respiratory syndrome coronavirus 2 (SARS-CoV-2) infection was found to induce tissue damage, thrombosis, red blood cell dysfunction and disrupt spermatogenesis through the oxidative stress pathway [144,145]. Respiratory syncytial virus (RSV)-induced oxidative stress is also thought to play a pivotal role in the pathogenesis of RSV-associated lung inflammatory disease [146]. Furthermore, chronic infection by hepatitis C virus (HCV) can cause liver inflammation (hepatitis), and HCV is associated with oxidative stress that results in increased levels of ROS [147]. As either the virus or viral PAMPs are recognized by RLRs, it is interesting to know how mutual regulations among virus, ROS and RLRs influence the progression of inflammatory diseases triggered by chronic viral infection. Therefore, it is highly advisable that more researches are needed to investigate the functions and molecular mechanisms of ROS-dependent activation of RLRs signaling and RLR-mediated ROS production in response to viral infection, which will provide potential therapeutic strategies for the prevention and treatment of inflammatory diseases triggered by chronic viral infection.

## Figures and Tables

**Table 1:** Therapeutic targets for suppressing inflammatory diseases based on the correlations between ROS regulation and PRRs-mediated signaling pathways.

Inhibitors	Class	Targets	Function	Reference
Therapeutic targets for inflammatory vascular diseases				
N-acetyl cysteine (NAC)	antioxidant	TLR2/TLR4	antioxidants treatment reduced TLR2/4 expression and downstream inflammatory biomediators	[98]
P-Coumaric Acid (P-CA)	hydroxycinnamic acid	TLR4	PCA alleviates experimental diabetic nephropathy	[99]
NAC	antioxidant	NLRP3 inflammasome	NAC suppressed NLRP3 inflammasome activation and improved HHcy-induced atherosclerosis	[100]
NAC	antioxidant	NLRP3 inflammasome	NAC diminished both inflammasome activation and inflammatory cytokine maturation, and subsequently pyroptotic death induced by nicotine	[101]
Peperomin E (PepE)	antioxidant	NLRP3 inflammasome	PepE inhibited atherosclerosis development in HFD-fed ApoE^−/−^ mice	[102]
EUK134	ROS scavenger	NLRP3 inflammasome	EUK134 decreased the MI/R-induced interaction between Txnip and NLRP3, and lowered MI/R-induced IL-1β mRNA and protein expression	[103]
Mito-TEMPO	mitochondrial ROS scavenger	NLRP3 inflammasome	Mito-TEMPO mitigated mROS levels and inhibited TMAO-induced activation of the NLRP3 inflammasome	[104]
Therapeutic targets for inflammatory neurological diseases				
Curcumin	polyphenolic compound	TLR4	Curcumin exerted anti-inflammatory action in MPP^+^-induced astrocytes	[106]
Tomentosin	active compound	TLR4	Tomentosin reduced behavior deficits and neuroinflammatory response in MPTP-induced Parkinson’s disease	[107]
Mito-TEMPO	mitochondrial ROS scavenger	NLRP3 inflammasome	Mito-TEMPO attenuated NLRP3 inflammasome activation	[109]
Astragaloside IV	active compound of *Astragalus membranaceus*	NLRP3 inflammasome	Astragaloside IV protected dopaminergic neuron from neuroinflammation and oxidative stress	[110]
Therapeutic targets for inflammatory bowel diseases				
Xi Lei San	Chinese patent medicine	NLRP3 Inflammasome	Xi Lei San ameliorated IBD by inhibiting NLRP3 inflammasome, autophagy and oxidative stress	[120]
Dimethyl fumarate (DMF)	Nrf2 activator	Nrf2 and NLRP3 inflammasome	DMF ameliorated DSS-induced murine experimental colitis	[121]
3-(2-oxo-2-phenylethylidene)-2,3,6,7-tetrahydro-1H-pyrazino-[2,1-a] isoquinolin-4(11bH)-one	Nrf2 activator	Nrf2 and NLRP3 inflammasome	Compound 1 protected against DSS-induced colitis	[122]
Curcumin	polyphenolic compound	NLRP3 inflammasome	Curcumin alleviated DSS-induced colitis	[123]
Procyanidin	antioxidant	NLRP3 inflammasome	Procyanidin attenuated DSS-induced experimental colitis	[124]
lactoferrin-modified liposomes (LF-lipo) containing Patchouli alcohol	active compound of Chinese herb patchouli	NLRP3 inflammasome	LF-lipo showed improved therapeutic efficacy in a DSS-induced colitis murine model	[125]
Melatonin	hormone	Intestinal microbiota, TLR2/TLR4	Melatonin mitigated “restraint stress”-induced colonic microbiota dysbiosis and intestinal inflammation	[134]
Walnut oil	dietary supplement	NLRP3 inflammasome, gut microbiota	Walnut oil alleviated DSS-induced colitis in mice	[135]
